# Fish Responses to Alternative Feeding Ingredients under Abiotic Chronic Stress

**DOI:** 10.3390/ani14050765

**Published:** 2024-02-29

**Authors:** Julieta Sánchez-Velázquez, Guillermo Abraham Peña-Herrejón, Humberto Aguirre-Becerra

**Affiliations:** 1 Facultad de Ingeniería, Campus Amazcala, Universidad Autónoma de Querétaro, El Marqués 76265, Querétaro, Mexico; julieta.sanchez@uaq.mx; 2 Centro de Investigación y Desarrollo Tecnológico en Materia Agrícola Pecuaria Acuícola y Forestal (CIDAF), Facultad de Ingeniería, Universidad Autónoma de Querétaro, Campus Concá, Arroyo Seco 76410, Querétaro, Mexico; guillermo.pena@uaq.mx; 3 Cuerpo Académico de Bioingeniería Básica y Aplicada, Facultad de Ingeniería, Campus Amazcala, Universidad Autónoma de Querétaro, El Marqués 76265, Querétaro, Mexico

**Keywords:** alternative feeding, chronic stress, cortisol, environmental stressors, animal welfare

## Abstract

**Simple Summary:**

Environmental factors are considered abiotic stressors when they negatively affect the integrity and welfare of fish. In aquaculture, many stressors occur under intensive fish farming (e.g., poor water quality, handling, crowding, and transportation). Abiotic chronic stress results in higher mortality and disease incidence, lowering fish growth and affecting aquaculture production systems’ performance. Feeding strategies can alleviate abiotic chronic stress in fish. Novel ingredients possess nutrients that confer antioxidant potential, improve the immune system, and reduce cortisol in blood plasma, the primary hormone in stress conditions. This management of chronic stress by alternative feeding is a sustainable form of obtaining food for human consumption, considering the animal welfare in intensive production systems. Alternative ingredients such as animal by-products, bacteria, fungi, and insect meal are described and discussed in this review as an alternative to mitigate fish chronic stress in aquaculture.

**Abstract:**

Aquaculture has become one of the most attractive food production activities as it provides high-quality protein for the growing human population. However, the abiotic chronic stress of fish in intensive fish farming leads to a detrimental condition that affects their health and somatic growth, comprising productive performance. This work aims to comprehensively review the impact of alternative and novel dietary protein sources on fish somatic growth, metabolism, and antioxidative capacity under environmental/abiotic stressors. The documental research indicates that ingredients from rendered animal by-products, insects, bacteria as single-cell proteins, and fungal organisms (e.g., yeast, filamentous fungus, and mushrooms) benefit fish health and performance. A set of responses allows fish growth, health, and survival to remain unaffected by feeding with alternative ingredients during chronic environmental stress. Those ingredients stimulate the production of enzymes such as catalase, glutathione peroxidase, and selenoproteins that counteract ROS effects. In addition, the humoral immune system promotes immunoglobulin production (IgM) and cortisol plasmatic reduction. Further investigation must be carried out to establish the specific effect by species. Additionally, the mixture and the pre-treatment of ingredients such as hydrolysates, solid fermentations, and metabolite extraction potentialize the beneficial effects of diets in chronically stressed fish.

## 1. Introduction

Abiotic stressors are environmental factors that affect fish growth, health, and survival, resulting in decreased production, profit, and product quality. Abiotic stress requires special attention in aquaculture [1,2,3]. Fish cells, tissues, and organs are negatively affected when any abiotic factor, such as oxygen concentration, temperature, or salinity, is far from optimal [4,5]. A stress response occurs when the homeostatic equilibrium of an organism is altered due to intrinsic or extrinsic stimuli that trigger the General Adaptation Syndrome (GAS). The GAS is a three-phase cascade of physiological responses: (1) Activation of the adrenergic system through sensory organs to increase plasma adrenaline and noradrenaline (catecholamines) [6]. (2) Hypothalamic–Pituitary–Interrenal (HPI) axis activation for plasma cortisol liberation, which, together with catecholamines, promote physiological modifications that include metabolic, hydromineral, hematological, immunological, and structural changes to mobilize energy resources [7]. In this phase, the stress responses are reversible, and the fish may return to a healthy pre-stress condition [8]. (3) The exhaustion phase results from continuous stress [2], where the energy and body resources meet the energy demand associated with the stress instead of being used in vital life processes, such as reproduction and growth, with detrimental consequences [9].

The stress response depends on fish adaptability, species, and stressor persistence or duration, which distinguishes acute from chronic stress [3,10,11,12]. Acute stress is a low-stress situation that lasts for a short time (minutes to hours) and may have stimulating, beneficial (eustress), or compensatory responses. In contrast, chronic stress lasts for days, weeks, or months and overstimulates coping responses that can negatively affect organisms (distress) [13,14]. Fish adaptability relates to regulatory mechanisms that compensate for the stressor effects [15]. Chronic stress must be considered a severe problem in aquaculture, which can be mitigated through sustainable management strategies (e.g., feeding and water quality control) that improve fish performance and welfare [16].

Dietary interventions have only been developed to manage acute stress in fish rather than chronic stress [17,18,19,20]. Feeding strategies to mitigate the effects of stress in aquaculture are explained by Kumar et al. (2015) and classified into nonchemical (biological) and chemical. Nonchemical methods involve environmental management (i.e., temperature, dissolved oxygen, ammonia, stocking density), while chemical methods include dietary supplementation [21]. Ciji and Akhtar (2021) classified feeding additives into nutritional (protein, amino acids, fatty acids, phospholipids, vitamins, minerals, carotenoids, nucleotides) and non-nutritional (biological products: prebiotics, probiotics, bacterial derivates, animal and plant extracts, yeast and its derivates, lactoferrins, micro/macro-algae, and polysaccharides; synthetic chemicals: propylene glycol, organic acids, clay additives, levamisole) [22].

Aquaculture nutritionists are developing diets with alternative ingredients to replace fishmeal with animal by-products, microalgae, bacteria, fungi, and insects. These inputs contain the required protein level for fish and nutrients such as antioxidants or immunostimulants, allowing the formulation of specific diets to manage chronic stress. However, there are few studies on the effect of alternative ingredients in aquaculture diets on fish’s health, growth, performance, and welfare as a possible strategy for alleviating chronic stress. Therefore, this article aims to review the use of dietary intervention in chronic stress under abiotic factors. First, the results from scientific evidence about the physiological, metabolic, and growth performance under abiotic chronic stress and dietary interventions with additives are summarized. Then, alternative ingredients such as animal by-products, bacteria, fungi, and insect meal are described and discussed to help cope with the environment or some abiotic stressors in fish under experimental conditions. Finally, a perspective on the feeding strategies to mitigate chronic stress in aquaculture is discussed.

## 2. Abiotic Chronic Stress Response and Feeding Intervention

The stress response of fish is a set of changes in their organism that neutralize the stressor to regain homeostatic equilibrium [23]. Many authors have pointed out several abiotic stressors and their harmful effects on fish., e.g., high-salinity and high-temperature stress on African catfish (*Clarias gariepinus*) [24], netting, confinement, flashlight, salinity and hypoxia challenges on European sea bass (*Dicentrarchus labrax*) [25], salinity or seawater culture on milkfish (*Chanos chanos*) [16], and chronic hypoxia stress on Nile tilapia (*Oreochromis niloticus*) [26]. However, authors do not use the term “abiotic stressor”; they usually use “environmental stressors”, “husbandry stressors”, and “water quality that includes physical or chemical factors” (Table 1). This review uses the term “abiotic stressor” to refer to any environmental factor negatively impacting fish.

The negative impact of chronic stress caused by abiotic factors includes (1) somatic growth, (2) metabolism, and (3) oxidative stress. A search of scientific studies on chronic stress in fish due to abiotic factors was performed to understand how dietary interventions modulate this condition. Two groups of keywords were used: (1) “chronic stress”, “feed”, “diet”, “aquaculture”, and (2) “chronic abiotic stress”, “feed”, and aquaculture between 2017 and 2023. A total of 15,060 and 2240 articles were found, respectively. Articles in which nutritive additives (e.g., amino acids, fatty acids, phospholipids, vitamins, and minerals) were used in diets with positive results in mitigating chronic stress on fish under abiotic stress were also considered (Table 2).

### 2.1. Somatic Growth

In aquaculture, faster fish growth is one of the most critical selection traits [30]. Stress is one of the most important physiological factors affecting growth. Several biotic and abiotic stressors (e.g., routine hatchery practices such as handling and sorting, poor water quality, and overpopulation) suppress growth [30]. Fish growth is the increase in length and weight, where muscle contributes over half of the body mass; thus, changes in muscle size are of primary importance to growth. Few studies have addressed the stress effect of cortisol stimulation on fish muscle function. Most have focused on the liver as the metabolic target for stress adaptation and cortisol action. Growth hormones (GHs) directly control somatic growth through receptors on the sarcolemma muscle and indirectly by initiating the production and release of insulin-like growth factor (IGF) in the liver and peripheral tissues [31]. IGF is a somatomedin produced in the liver that regulates the growth and metabolism of fish. The IGF system comprises IGF-I, IGF-II factors, several receptors IGF-IR and IGF-IIR, and six binding proteins (IGFPs). The IGF system is one of the central pathways that regulate protein synthesis in skeletal muscle [31]. The IGF-I and IGF-II factors bind to IGF-IRs (IGF receptors), which activate the PI3K-Akt-TOR pathway. This action results in translation and protein synthesis to form muscle by stimulating cell proliferation, differentiation, hypertrophy, and inhibiting muscle atrophy [32]. However, cortisol inhibits protein synthesis by decreasing the myogenic gene expression and reducing the action of the GH–IGF axis [30]. Therefore, blocking cortisol in stressed fish mitigates adverse effects on muscles.

Tryptophan (Trp) is an essential amino acid metabolized into serotonin, melatonin, and kynuenine [33]. Serotonin directly blocks cortisol [34], while kynurenine provides energy substrates, such as Acetyl CoA and pyruvate, that enter the Krebs cycle to respond to the energy demand under stress [35]. In this sense, *Oncorhynchus mykiss* fed with the Trp diet significantly reduced stress-induced plasma cortisol [36]. *Labeo rohita* fed with a 1.4% Trp-enriched diet and exposed to temperature and salinity stress significantly increased the weight gain to 21.46 ± 1.00% compared to fish fed without Trp under both stress conditions with 12.72% [37]. Dietary Trp serves not only for synthesizing neurotransmitters but also for body protein deposition. Trp conference resistance and promote fish growth despite stress. Herrera et al. (2020) observed the maximum values for final weight when meager *Argyrosomus regius* was fed with a Trp-supplemented diet and subjected to chronic stress (Table 2) [38]. Additionally, Trp supplementation counteracts the stress-induced elevations of plasma cortisol in rainbow trout (*Oncorhynchus mykiss*) under high stocking density [36].

Phenylalanine (Phe) is an essential amino acid metabolized through two metabolic pathways: Tyrosine (Tyr) oxidation and transamination to phenylpyruvate. Tyr is a direct precursor of the catecholamine hormone, a compound related to stress response, and the hydroxyphenyl pyruvate as part of the energy metabolism [39]. Phe and Tyr provide the energy demand when fish are under acute or chronic stress, but they also boost fish growth under chronic stress and reduce stress markers such as cortisol [40]. Under chronic stress, Phe enriched diet in Gilthead seabream (*Sparus aurata*) decreases cortisol levels but decreases fish growth. Authors suggest an unbalanced diet (amino acid excess) results in malnutrition and reduced growth [41]. Including amino acids guarantees positive effects on fish growth during chronic stress.

### 2.2. Metabolic and Cellular Markers

The stress response involves changes in metabolic pathways [42]. Cortisol modulates energetic metabolism. Under stress conditions, the increase in plasma cortisol promotes protein, lipids, and glucose mobilization in the skeletal muscle, which is the principal target of cortisol [43]. A critical metabolic role for cortisol during stress is elevating circulating glucose levels by increasing hepatic gluconeogenesis to fuel the increased energy demand, especially during environmental fluctuation and stress response [44]. The preceding is accompanied by enhanced muscle protein degradation, which increases the availability of amino acids as substrates for gluconeogenesis in the liver [45]. In addition, this provides energy to organs, such as the brain, to overcome the stressor. Cortisol exerts a critical control over skeletal muscle glucose metabolism, inhibiting glucose uptake and utilization and glycogen synthesis [43].

Cortisol in the blood provides information on the stress response. The average resting levels of plasma cortisol in fish can be as low as 13.8 nM, while fish with chronically activated stress response can have a resting level greater than 27.5 nM [46]. However, cortisol cannot be just related to negative stressors; not all cortisol fluctuations indicate distress. Thus, it is essential to differentiate the baseline from a standard range of stress-related concentrations [29]. Cortisol is a hyperglycemic hormone as it causes the elevation of plasma glucose, providing fish with energy to escape or cope with an adverse situation [47]. Elevation in plasma cortisol stimulates glycogenolysis, i.e., the conversion of glycogen stored in the tissue to glucose released into the blood. Increased plasma glucose in the blood is a relatively slow response to a stressor, and pre-stress plasma glucose levels are usually between 3.7 and 4.6 mM [46].

Lactate is produced by anaerobic ATP production (glycolysis) when oxygen is unavailable for cells during aerobic metabolism. The peak of plasma lactate during stress events ranges from 6.4 to 13.3 mM (normal condition 3 to 5 mM). Cortisol in plasma stimulates anaerobic metabolism that results in lactic acid accumulation. In normal conditions, most lactate is produced by the erythrocytes; however, during intense physical activity, the muscle produces large amounts of lactate because of insufficient muscle oxygenation [47]. Salamanca et al. (2022) showed the reduction of lactate in *Sparus aurata* subjected to chronic stress and fed with a Phe-enriched diet [41]. Additionally, Herrera et al., 2020 showed the same effect of Trp-enriched diets in *Argyrosomus regius* [38] (Table 2). Finally, Longbaf Dezfouli et al. (2019) showed the reduction of plasmatic glucose, increased weight gain, final weight, and feed intake in *Lates calcarifer* subjected to freshwater as an abiotic stressor and including magnesium and selenium nanoparticles as alternative ingredients in the fish diet [48].

### 2.3. Oxidative Stress

Organisms subjected to stressors undergo physiological changes and activation of biochemical pathways mediated by the neuroendocrine system. Additionally, cellular stress includes oxidative stress caused by overproduced reactive oxygen species (ROS) in fish [49,50]. Oxygen is the primary biological acceptor of electrons with a vital role in cellular functions. However, it contributes to the undesirable formation of ROS, such as superoxide, hydrogen peroxide, and hydroxyl radical. ROS can be classified as free radicals or non-radicals [5]. Organisms have enzymatic and non-enzymatic antioxidant defenses to minimize the damaging effects of ROS. Enzymatic antioxidants include superoxide dismutase (SPD), catalase (CAT), glutathione peroxidase (GPx), glutathione reductase, and glutathione-S-transferases. In contrast, non-enzymatic antioxidants are vitamin E, vitamin C, β carotene, vitamin A, glutathione, flavonoids, thiols, coenzyme Q, and uric acid [5].

Cellular defense pathways in fish are triggered by oxidative stress due to acute and chronic unfavorable water conditions. The primary cellular markers during oxidative stress include the nuclear factor erythroid 2 (NRF2), HIF1α, heat shock protein 70 (HSP 70), tumor necrosis factor-alpha TNFα, and nuclear factor kappa-light-chain-enhancer of activated B cells [4]. Kulczykowska (2019) suggests that mucosal skin markers activate during oxidative stress in fish (e.g., melatonin (Mel) and its active metabolites) [51].

Research supports that dietary intervention with certain nutrients modulates chronic stress. Harsij et al. (2020) proved that diets enriched with selenium, vitamin C, and E for *Oncorhynchus mykiss* under ammonia exposure had positive effects on the antioxidant response as the levels of aspartate aminotransferase (AST), alanine transaminase (ALP), and alkaline phosphatase (ALT) were decreased [52]. Authors suggest that selenium protects fish against stress by forming seleno-proteins such as GPx and thioredoxin reductase. Additionally, juveniles of Wuchang bream (*Megalobrama amblycephala*) under ammonia stress fed with dietary selenium, yeast, and polyphenols showed decreased levels of cortisol, TNFα, IL-1β, GPx, and increased CAT [53].

Agavine, a bioactive compound from agave agro-industrial wastes, effectively functioned in juvenile Nile Tilapia (*Oreochromis niloticus*) stressed by high-density stress (63 kg/m^3^) for 20 days. Stressed fish fed with agavine presented low plasmatic cortisol and high levels of SOD and CAT, promoting the antioxidant effect of fructants that produce fermented metabolites [54]. Cruz-Marín et al. (2023) used inulin, a nondigestive additive, in native fish tropical gar (*Atractosteus tropicus*) larvae. Fish fed with 2.5% inulin showed diminished intestinal activities of GPx, SOD, and CAT, which resulted in improved antioxidant status. Additionally, the larvae survival increased as the concentration of inulin increased [55]. In Nile Tilapia (*Oreochromis niloticus*), similar effects of dietary inulin were observed under 16 hypersaline-stressed conditions for eight weeks, similar to a chronic state as CAT and GAST diminished in the gut [56].

More examples of how dietary interventions with certain additives modulate chronic stress are shown in Table 2.

### 2.4. Immune Response

There is much information about the effects of additives on fish immune responses to prevent health care by dietary manipulation. This strategy is supported by substantial evidence that nutrition is an essential modulator of the fish immune system [57]. In this sense, in aquaculture practices, knowing those dietary additives that positively affect stressed fish at a chronic level is crucial and allows the possibility to cope with any threat. Acute stress stimulates the fish immune response by showing an activating phase that significantly enhances innate responses, such as recruiting subsets of immune cells and the presence of cytokines proinflammatory such as interleukin-8 (IL-8), C-C motif chemokine ligand 2 (CCL2), interleukin -2 (IL-2), interferon-gamma (IFN-γ), and TNF-α [6]. At the same time, cortisol in the blood is often observed under acute stress, and in conjunction with IL-1β and TNF-α is a critical mediator of acute inflammatory reactions to counteract external challenges [58]. After this process, anti-inflammatory cytokines down-regulate inflammation. However, chronic stress and its primary signaling mediator, cortisol, have traditionally been understood to have an overall suppressive effect on fish immunity, reducing antibody production, leukocyte mitosis, phagocytosis, and leukocyte trafficking. Thus, the continuous presence of cortisol promotes the expression of proinflammatory cytokines such as IL-1β, TNFα, and IL-6 [6,59,60,61].

Cytokines play an essential role in the innate adaptive and immune response. A series of sensitive molecular reactions regulate relative signal transduction from environmental stress to inflammatory response. SOCS (suppressor of cytokine signaling) family members function as feedback inhibitors of cytokine signaling by inhibiting various signal transduction pathways. SOCS1, the prototype of SOCS members, is deeply involved in negative regulations of cytokines through the Janus kinases (JAKs)—signal traducers and activators of the transcription (STATs) pathway. Elevated cortisol due to stress response can inhibit cytokine signaling by binding to glucocorticoid receptor (GR), suppressing the JAK/STAT3 pathway, enhancing the SOCS1 expression, and decreasing proinflammatory cytokine production [59]. Then, the stress response is mediated by the close interaction of hormones and cytokines [60]. Dai et al. (2023) noticed that 21 days of chronic stress in *Carassius gibel* promoted systemic inflammation, and fish showed a significant increase but low fold changes in IL-1β, IL-6, and IL-8 in the trunk kidney, spleen, intestine, and liver. The increased amplitude of all detected proinflammatory cytokines remained relatively low, suggesting sustained low-grade inflammation. Midgut samples manifested morphological changes, including damage in obliterated villi, decreased number of goblet cells, and loosened submucous layer. Microbiota also changed, reducing *Bifidobacterium*, *Lactobacillus*, *Faecalibacterium*, and *Flavobacterium* [62]. These results showed the detrimental effects of chronic stress in fish, the importance of microbiota, and that any damage in the gut might directly affect fish’s immune status [63]. In the same way, low-grade inflammation differs from normal inflammation in that there are no typical signs of inflammation. However, it is also similar in involving the same inflammatory mediators and signaling pathways. Having unraveled the communication pathways between the brain and the gut, the gut microbiota has emerged as an essential component affecting all neuroimmune endocrine pathways. Microorganisms are vital in controlling stress and the gut–brain axis under stress conditions [64]. Increasing attention has been paid to the microbiota–gut–brain (MGB) axis, and its potential role in the immune system is a crucial communication pathway between the gut microbiota and the brain [63].

Nutritionists are working on immunomodulation to promote intestinal microbiota by enhancing protection through dietary supplementation using nondigestive ingredients. Prebiotics are non-digestible food ingredients that beneficially affect the host by selectively stimulating the growth and activity of health-promoting bacteria in the intestinal tract [65]. Fructooligosaccharides (FOS), mannooligosaccharides (MOS), and inulin are the most studied prebiotics in fish, while short-chain Fructooligosaccharides (scFOS), galactooligosaccharides (GOS) and xylooligosaccharides (XOS) have been much less studied [63,66]. Asencio-Alcudia et al. (2023) demonstrated the protective role of dietary α-Tocopherol, the most active form of vitamin E, in Longfin Yellowtail (*Seriola rivoliana*). Fish fed with 500 mg/kg α-Tocopherol show an enhanced activity of the anti-inflammatory process by over-expressing immune system genes IL-10 in the spleen due to the immunomodulation effect of additives [67].

Zhang et al. (2020) showed that administering FOS in chronically stressed mice diets can prevent intestinal barrier damage and neuroinflammation by expressing IL-10 anti-inflammatory cytokine, while the expression of TNF-α decreases in the gut [68]. The protective effect of FOS supplementation was demonstrated by reducing the pro-inflammatory response in goldfish (*Carassius auratus*) subjected to different LC50 concentrations of triphenyltin for short periods. Fish fed with FOS showed significant suppression levels of pro-inflammatory cytokines such as TNF-α, IL-6, and IL-1β [66]. *Megalobrama amblycephala* fed 0.4% FOS after six hours of 10 mg/L ammonia exposure presented an increment of nonspecific immunity through the lysozyme activity [69].

A mixture comprising live microorganisms and substrates selectively utilized by host microorganisms was an effective dietary symbiotic supplementation, as evaluated by Singh et al. (2019) [70]. *Bacillus circulans* PB7 (BCPB7) and FOS were proposed to improve the immune response of juveniles *Labeo rohita* under persistent stress to low pH for 60 days. Lysozyme and antioxidant activity were enhanced in fish fed with the synbiotic treatment compared to those organisms fed only with probiotic BCPB7 or FOS [71] (Table 2).

**Table 2:** Additives used in diets with positive effects on mitigating abiotic chronic stress on fish.

Nutrient	Supplementation	Specie	Chronic Stress Factor	Observation	Reference
Tryptophan	*Argyrosomus regius*	Crowding and netting stress for four months	0.25% Trp	No affects body weight, maintain glucose, lactate, and cortisol level ↓ Cortisol in mucus skin ↓ Plasma lactate	[38]
Tryptophan	*Oncorhynchus mykiss*	High density for 70 days	5 g Trp per kg diet	↑ Lysozyme ↑ Bactericidal activity ↓ CAT and MDA	[33]
Methionine, Lysine, Tryptophan, Threonine	*Oncorhynchus mykiss*	For six weeks, two times per week, handling stress 30 s of chasing, followed by capture in nets, removal from tanks, and 30 s of air exposure	0.17% DL-Methionine 0.81% L-Lysine sulfate 0.22% L-Tryptophan 0.20% L-Threonine		
Phenylalanine	*Sparus aurata*	Confinement and netting/chasing stress 5 min 3 times a day	5% Phe	↓ Plasma lactate	[41]
Magnesium and selenium nanoparticles	*Lates calcarifer*	Freshwater	4 mg NanoSe/kg diet 500 mg NanoMg/kg diet	↑ Final weight, weight gain, SGR, feed intake ↑ IgM and ACH50 ↑ ALT, AST ↓ Glucose	[48]
Nucleotides	Hybrid stripe bass *Morone chrysops* × *Morone saxatilis*	Salinity of 15 g/L for four weeks	0.5% de Adenosine 5′-monophosphate	↓ Blood glucose ↑ Weight gain ↑ Lysozyme activity, anti-protease activity	[72]
Vitamin E and NanoSe	*Oncorhynchus mykiss*	Dense stocking density (80 kg/m^3^) 60 days	500 mg/kg Vit E and 1 mg/kg nano Se	Final Weight ↑ SGR ↑ FCR ↑ Cortisol ↓ Lactate ↓ ALT ↓ AST ↓ ALP ↓	[73]
NanoSe, Vitiamin C and E	*Oncorhynchus mykiss*	Sublethal concentration of ammonia exposure (0.024 mg/L)	0.2 mg/kg NanoSe, 200 mg/kg Vitamin C and 60 mg/kg Vitamin E	↑ final weight, ↓ FCR ↓ AST and ALP ↑ ALT, TAG, IMg Lysozyme	[52]
DHA/EPA	*Salmo sala*	Three weeks with an unpredictable chronic stress (UCS) protocol	25 g/kg EPA 14.2 g/kg total fatty acids	↑ weight gain ↓ mucosal fold height, enterocyte height, and vacuolization Gene expression related to environmental information processing	[74]
n-6/n-3	*Salmo salar*	Hypoxia 3 times per week, during four weeks	Diet 6 46.7% LA 18:2n-6 0.1%ARA 20:4n-6 3.1%18:3n-3 2.0% EPA 20:5n-3 1.8% DHA 22:6n-3 3.8% EPA + DHA	Suppressed cortisol response. ↑ Level of eicosanoid PGD2 in liver ↑ level or leukotrienes LTB4 1 h after acute stress, LTB4 was the eicosanoid with the highest concentration before the acute stressor. ↓ IGF-1 was significantly lower	[75]
*S. cerevisiae*	Nile tilapia (*Oreochromis niloticus*)	Heat and hypoxia, Dynamic heat stress 0.01 °C per min up to 40 °C Static heat stress from 40 °C to 28 °C for 90 min Exposure to glyphosate and/or malathion Hypoxia stress all consume of oxygen (0 mg/L) for 24 h	50–70% *S. cerevisiae*	↑ survival	[76]
Selenium yeast supplementation	Nile tilapia (*Oreochromis niloticus*)	60 days against the harmful effects of glyphosate and/or malathion chronic toxicity	Selenium yeast supplementation 3.3 mg/kg diet (2.36 mg/kg selenomethionine and 0.94 mg organic selenium)	↑ survival ↑ growth ↑ SGR, ↓ FCR ↑ protection for free radicals	[77]
Spent oleaginous yeast	Juvenile red sea bream (*Pagrus major*)	Low salinity water (0.2%) The test was terminated when all the fish died.	2.5%, 25 g/kg supplement spent oleaginous yeast	↑ FW, SGR ↓ FCR ↑ SOD, GPx, IgM, and Lysozyme activity Maintain values of MDA	[78]
Dietary selenium yeast and tea-polyphenols	Juvenile Wuchang bream (*Megalobrama amblycephala*)	Ammonia stress 22.5 mg/L ammonia	Dietary selenium yeast and tea-polyphenols	↓ Cortisol, TNFα, IL-1β, ↓ GPx ↑ CAT	[53]
*Bacillus circulans* PB7 (BCPB7) and Fructoligosaccharide	Juveniles *Labeo rohita*	Low pH for 60 days		↑ WG, SGR ↓FCR ↑ Lysozyme activity ↓ Cortisol ↓ HSP70	[71]
*B. coagulants*	Common carp (*Cyprinus carpio*)	Long-term exposure to Cd^2+^ 30 and 60 days	2.0 × 10^8^ CFU/g of *B. coagulants* in diet	Activation of Nrf gene family for resistance to oxidative stress and immune response	[79]
*Clostridium autoethanogenum*	Largemouth bass (*Micropterus salmoides*)		*Clostridium autoethanogenum*		[80]

## 3. Discussion

### 3.1. Feeding Intervention with Alternative Ingredients in Fish

Considering the issues that arise from the scarcity of suitable protein sources (e.g., fishmeal in animal farming) in the aquaculture industry, increasing prices of feeding, and environmental concerns, farmers are forced to prove alternative protein-rich ingredients to be replaced in conventional fish meal [81]. However, in the frontier of science in aquaculture, alternative ingredients are considered to replace fish meal as they confer fish health benefits during environmental changes that cause chronic stress. In this section, we discussed using alternative ingredients such as animal by-products, bacteria, fungi, and insects’ meals in fish feeding under chronic stress and their benefits in fish growth performance, metabolic and cellular markers, and oxidative stress.

### 3.2. Animal By-Products Meal

Animal by-product meal (ABPM) consists of the discarded parts of farmed animals and represents a viable alternative to protein sources to replace FM in aquafeed [82]. ABPM is the group of ingredients that include meat and bone meal (MBM), bone meal, hydrolyzed feather meal, and poultry by-product meal [83,84]. Products from the non-ruminant and poultry processing industry suffer physicochemical transformation to obtain high aggregate value ingredients [83,84]. A disadvantage of ABPM as an FM protein replacement is reduced digestibility and feed efficiency at high dietary inclusion levels [85]. ABPM biotransformation with yeast or enzymes to obtain hydrolysates that increase digestibility and feed efficiency is under research [85]. ABPM is a good protein source to replace FM [86]. However, further studies are necessary to establish an adequate inclusion to avoid adverse effects on growth and improve fish condition under chronic stress abiotic. The work with positive results in fish related to stressful conditions and the partial substitution of FM with MBM occurs under acute stress. Zare et al. (2023) recorded netting results as a stressor and FM replacement with MBM and their interactions on growth, blood chemistry, immune responses, antioxidant system, and stress response in the ornamental fish *Astronotus ocellatus* [87].

### 3.3. Bacteria

The growing bacterial protein industry has gained much attention as an alternative protein source for animal diets, as no land to grow crops is used [88]. Bacterial protein meal (BPM) contains about 70% crude protein, 10% crude lipid (dry matter), a well-balanced amino acid profile, and vitamins similar to fish. BPM improves some aquatic animals’ digestive capacity, meal taste, and immune function [80]. BPM that exert beneficial effects on fish health are *Lactobacillus*, *Shewanella*, and *Bacillus* [89].

In Salmo salar, BPM replaced up to 25% of the amino acids from FM, but fish growth performance and survival were negatively affected when reaching 50% of BPM. Tang et al., 2020 proved that dietary supplementation with a host-derived *Bacillus subtilis* strain in *Oreochromis niloticus* under hypersaline stress for eight weeks resulted in a *significantly* higher growth rate (4.8%/d) and weight gain (1400%). Regarding immune response, the brackish water and *B. subtilis* treatment presented the highest hemolytic complement (CH50) level and the lowest immunoglobulin M (IgM compared to the control. The presence of the complement system (CH50) is directly related to killing microorganisms. A complement system is a group of proteins that, when activated, lead to target cell lysis and facilitate pathogen microorganisms’ phagocytosis through opsonization during immune response [90], and the absence of IgM could be a response to severe injury by hypersaline stress. On the contrary, fish control treatment without *B. subtilis* feeding and freshwater or brackish water showed low levels of CH50 and high levels of IgM. Then, *B. subtilis* can be implemented as a dietetic strategy to help fish cope with stressful environments [91]. Lactic acid bacteria (LAB) have been widely used in aquaculture as feed-supplements probiotics. *Lactobacillus plantarum* is a probiotic that enhances fish growth, health, antioxidants, and immunity [92].

### 3.4. Fungal (Yeast, Filamentous Fungi, and Mushroom)

Fungal biomass is gaining attention due to the emerging need for suitable and sustainable novel feed ingredients for aquaculture. Fungi comprise a large amount of morphological diversity, from unicellular yeasts to large fruiting bodies [93]. Yeast protein biomass is a potentially sustainable ingredient because it converts low-value non-food products from the agricultural industry into high-value feed with less dependence on arable land and water [94]. Yeast composition presents approximately 50% of protein content, trace minerals, and vitamins, including B-group [95]. In searching for strategies to introduce yeast in fish diets, yeast hydrolysate (YH) has high protein content and nutrients and is listed in the Chinese “feed material catalog” [96]. Yuan et al. (2017) obtained significant results in juveniles of *Cyprinus carpio* (var. Jian) fed with a yeast hydrolysate-enriched diet (3% YH) by showing the fish’s highest final weight (178.60 ± 8.42 g). Fish subjected to biotic stress with *Aeromonas hydrophila* displayed a modulated inflammatory signal, indicating that an adequate replacement level may help avoid the inflammatory response [97].

Species such as crustaceans have also shown the functionality of YH-enriched diets to manage abiotic stress. Environmental stress triggers the overproduction of ROS, producing cell damage. In crustaceans, antioxidant defense, especially the specialized antioxidant enzymes SOD and GPx, plays a crucial role in eliminating ROS [98]. In response to abiotic stress, (Chen et al., 2020) described positive results in *Litopenaeus vannamei* fed for eight weeks with 0.5% YH (*Rhodotorula mucilaginosa*) and 0.1% *Bacillus licheniformis* (BL). In this work, shrimps subjected to a high ammonia level (150 g/L NH_4_CL into 100 L) and fed with a combination of 0.5 YH/0.1 BL and only 0.5% HY treatment (60%) showed the highest survival (80% and 60%, respectively). In addition, shrimps fed with a combination of YH and BL showed an enhanced antioxidant system, the levels of CAT, GPx, and SOD [99]. CAT, GPx, and SOD are the enzymes that reduce free radicals and prevent cellular damage. Jin et al. (2018) evaluated the effects of dietary YH and brewer yeast supplementations on growth performance and ammonia nitrogen stress resistance (24 h solution of 28 mg/L ammonia) of *Litopenaeus vannamei*. Shrimp fed with 1% yeast hydrolysate supplementation showed the highest final growth and the lower inflammatory-related gene expression of TNF-α and IL-1β in the intestine, and surprisingly, the levels of mTOR signal pathway genes were significantly up-regulated in those fed with 1% supplemented yeast hydrolysate [100].

#### 3.4.1. Filamentous Fungus

Filamentous fungi generate multicellular colonies, or mycelia, through hyphae cells’ extension and repeated branching [101]. When hyphae aggregate, more complex three-dimensional structures emerge. The most complex structures in the fungal kingdom are the multicellular sexual fruiting bodies, with distinct fungal tissues and multiple cell types [102]. Fruit bodies, including the familiar mushrooms of basidiomycetes, are multicellular reproductive organs. Mushroom formation involves the coordinated growth of millions of hyphae [102]. Filamentous fungi in the diet offer benefits to fish, including growth promotion, inhibition of pathogen colonization, improvement of nutrient digestion, and enhancement of reproduction. However, there are very few insights on chronic alleviation [103]. *Aspergillus* sp. V2 grown on vinasse can be used for fish feed. Proximate analysis revealed a total protein content of 34%, fat of 4.7%, ash of 15.8%, crude fiber of 4%, moisture of 0.3%, and carbohydrate of 45.2%, all within acceptable levels for commercial fish diets. Its composition is analogous to soybean meal, containing all the essential amino acids for fish feeding and permissible aflatoxin levels for feedstuff [104]. *Aspergillus niger* is a promising ingredient for formulating cost-effective shrimp feed. This fungus has been reported to produce numerous hydrolytic enzymes for degrading the limiting factors of plant proteins, such as antinutrients and fiber. *A. niger* enhances the quantity and quality of proteins and amino acids, especially methionine and lysine, helping to replace dietary fishmeal up to 80% in penaeid shrimps [105].

Dawood et al. (2020) used two levels of supplemented *Aspergillus oryzae* (ASP 1 × 10^6^ CFU/g and ASP 2 1 × 10^8^ CFU/g) in Nile tilapia (*Oreochromis niloticus*) under hypoxia, resulting in enhanced oxidative status, heat shock protein, and immune-related gene expression. The hypoxia stress was achieved by reducing the tank water volume until the symptoms in the dorsal fin were evident. Fish with both diets presented significantly higher levels of final body weight (61.40 ± 0.58 g with ASP and 63.25 ± 1 with ASP2) than fish control (56.77 ± 0.26). In terms of blood chemistry, antioxidant status, and immunological response after hypoxia, fish with supplemented diets showed lower glucose and cortisol concentrations (ASP 2 148 mg/dL and 0.65 mM/mL, respectively) in comparison with the fish control diet (160 mg/dL and 0.9 mM/mL). The enzymatic activity in fish fed with supplementation ASP 2 had higher levels of SOD (26 IU/L), GPX (25 IU/L), and lower MDA (14 nmol/mL), which are antioxidant defense network compounds, indicating reduced cell damage compared with fish control diet (SOD 21.5 IU/L, GPX 15.7 IU/L; MDA 17.5 nmol/mL) [106].

#### 3.4.2. Mushroom Meal

Macroscopic filamentous fungi that form large fruiting bodies are known as mushrooms [107]. Edible mushrooms are the higher fungi in the *Ascomycetes* and *Basidiomycetes* classes [108]. Great attention was given to the polysaccharides produced by numerous fungi, especially mushrooms, because of their various biological properties, such as antioxidant, antimicrobial, and immunostimulant activities [109].

Mushrooms have been consumed as a healthy nutritional food and medicine for decades due to their significant antioxidant, anti-inflammatory, anti-tumor, and immune-stimulating properties [110]. By-products of king oyster mushroom *Pleurotus eryngii* (KOME) and *Lactobacillus plantarum* (LP) were incorporated into the white shrimp *Litopenaeus vannamei* diet. Shrimp fed with 5 g/kg KOME and 1 × 10^8^ CFU/kg LP had significantly higher weight gain and total final weight than the control [110].

*Pleurotus ostreatus*, also known as the oyster mushroom, has anticancer, immunomodulatory, antiviral, antibiotic, anti-inflammatory, and cholesterol-lowering properties. A diet supplemented with this mushroom for *Oreochromis mossambicus* showed a positive effect on survival rate when fish were subjected to septicemia [111]. *Agaricus blazei* is an edible mushroom native to Brazil that has gained interest in various fields of medical research, as it has shown remarkable immunomodulating effects. The number of by-products and waste substrate from mushroom production has increased drastically as the annual global mushroom yield is estimated to be over 25 million tons. P.T. Lee, Wu, Tseng, Lu, and Lee described efforts to recycle and reuse waste mushroom compost, taking advantage of its immunomodulatory compounds, highlighting that waste mushroom substrate from *A. blazei* cultivation is a cost-effective feed additive for *Oreochromis niloticus* that protects fish from *S. agalactiae* infection [112].

A Taiwanese medicinal mushroom, *Antrodia cinnamomea* (AC), possesses triterpenoids, polysaccharides, superoxide dismutase (SOD), adenosine, succinic and maleic acid derivates, proteins, vitamins, nucleic acid, lectin, ergosterol, and lignin. The raw materials of this mushroom have bioactive compounds. Zebrafish were fed with an AC-supplemented diet for studying fish heat resistance (increased temperature from 28 °C to 39 °C in 100 min) and cold tolerance (decreased temperature from 28 °C to 11.5 °C in 100 min). Although this work describes acute stress conditions, the results for survival, growth performance, and immunology responses were improved. Fish fed with 10% AC presented almost 95% survival under heat and 80% under cold. After 30 days, the group of 10% AC/39 °C showed a significantly increased body weight gain (0.107 ± 0.018 g) compared to the 0.2% AC/39 °C group (0.086 ± 0.024 g). Fish fed with 10% AC presented the highest specific growth rate (1.084 ± 0.082%/day) compared to the 0.2% AC group (0.859 ± 0.075%/day) [113].

Enhancing the antioxidant defense status by supplementing with natural antioxidant-rich sources is the most convenient alternative to protect fish from stress. Disposing of undesirable mushroom parts, such as mushroom stalk waste (MSW), increases exponentially along with its production. As mushrooms are a rich source of polysaccharides and antioxidants, raw polysaccharides (RPs) from MSW were supplemented to fish feed to evaluate the protective effect on stress caused by pH fluctuation. The 5 and 10 g RP/kg feeds showed the highest survival for *Oreochromis niloticus*. Fish post-stress conditions in RP 5 g/kg feeds were improved, with enhanced SOD and CAT activity for pH 5.5, 8.5, and 10.5 during one week [114].

### 3.5. Insect

Insect meal has been suggested as a promising and natural alternative to FM and FO, as it contains a large number of amino acids, lipids, vitamins, minerals, and crude protein, where the latter represents between 50% and 82% of its dry mass, depending on the insect species and processing method [115]. Given these promising features, several types of insects, such as Silkworm (*Bombyx mori*), black soldier fly (*Hermetia illucens*), Housefly (*Musca domestica*), Yellow mealworm (*Tenebrio molitor*), Lesser mealworm (*Alphitobius diaperinus*), House cricket (*Acheta domesticus*), Banded cricket (*Gryllodes sigillatus*), and Jamaican field cricket (*Gryllus assimilis*) have been considered to elaborate meal [116]. Although the impact of insect meal on chronic stress has not been studied, some investigations have proven benefits in fish health. For example, an immunostimulant effect was described in rainbow trout *Oncorhynchus mykiss* with black solider fly and poultry by-product combination [117], in Pacific white shrimp (*Litopenaeus vannamei*) [118] and mandarin fish (*Siniperca scherzeri*) with Yellow mealworm (*Tenebrio molitor*) [119], in Atlantic salmon (*Salmo salar*) with black solider fly [120], and Nile tilapia (*Oreochromis niloticus*) with black soldier fly larvae [121]. Taufek et al. (2018) observed the effect of the cricket meal diet (*Gryllus bimaculatus*) on African catfish (*Clarias garienpinus*). After 40 days with experimental diets, fish were subjected to bacterial stress with *Aeromonas hydrophila* [122]. The results showed that fish fed with 40% of *G. bimaculatus* presented elevated levels of white blood cells (19.2 ± 0.13 × 10^3^ mm^3^) and lower with FM (13.6 ± 0.86 × 10^3^ mm^3^), higher lysozyme activity (22.2 ± 2.54 U/mL) compared to FM (8.4 ± 1.16 U/mL), and more elevated globulin in serum (59 ± 6.08 mg/dL) compared to FM (51.5 ± 5.49 mg/dL). Moreover, after 12 days of *A. hydrophila* infection, fish fed with cricket meal presented a 66.7% relative survival percentage, indicating a positive immunostimulant effect.

BSFL meals have been tested in different studies with different inclusion levels, ranging from 25% to 100%, meal quality, fish species, and diet formulations. Kortner et al. (2020) reviewed the effect of an insect meal-based diet (60% BSFL) on the gut health of Atlantic salmon in freshwater and seawater conditions. The results showed that fish from freshwater (pre-smoltification) presented gene expression related to immune tolerance (foxp3), stress response (hsp70), and detoxification activity (CYP1A1, MTA, SOD, AND CAT); additionally, no enterocyte steatosis in the proximal intestine was observed. Enterocyte steatosis is a metabolism disorder in enterocytes, which, in severe cases, may be accompanied by accumulations of lipidic materials inside the gut lumen [123]. The effect of BSFL meal was supplemented in a diet for juvenile rainbow trout (Oncorhynchus mykiss) reared at different stocking densities [124]. At 4.9 kg/m^3^, fish fed with a diet of 50 g/kg BSFL meal and 10 g/kg BSFL oil showed an increase in final growth (27.12 ± 0.42 g) compared to control diet (without supplementation 26.07 ± 0.42 g). In terms of plasma antioxidant metabolites, immune and stress-related parameters of rainbow trout at the same stocking density showed the highest values of IGF-I (450 pg/mL), SOD (9 ng/mL), IL-1β (21 pg/mL), Lyz (30 U/mL), and lower values of HSP-70 (7.9 ng/mL) and Cortisol (30 ng/mL).

### 3.6. Non-Conventional Plant Meal

The most important groups of seed crops used for food include cereals, pseudocereals, pulses, oilseeds, and nuts, with a loss or waste through the food chain. Seed loss refers to materials that do not reach consumers. In contrast, seed wastes are all those parts of the seeds not intended for human consumption, including hull, husk, or shattered cotyledon, often produced in dehulling and milling processes. This seed coating contains bioactive compounds [125]. Corn husk is part of the corn stover, which has the highest concentration of phenolic compounds with ferulic and ρ-coumaric acids [126]. Galeana-López et al. (2021) used a 25 g corn husk meal/kg diet in Nile Tilapia (*Orechromis niloticus*) exposed to hypoxia at 1.5 mg/L of oxygen dissolved for a short period of 5 h, resulting in a significant increase in CAT activity in tilapias exposed to stress [127].

One problem with employing plant sources as alternative ingredients in aquafeeds is the anti-nutritional factors that impede the digestive process in fish. A solution uses biological strategies that degrade these compounds, such as solid-state fermentation (SSF), which involves the microbial fermentation of a substrate where subsequent by-products may provide residual bioactive components [114]. Amaral et al. (2023) evaluated the effect of SSF on plant feedstuff (soybean, rapeseed, sunflower, and rice bran) fermented with *Aspergillus niger* in stressed European Seabass (*Dicentrarchus labrax)*. However, the results of fish-fed test diets subjected to different temperatures and salinity oscillation indicated that fermented products did not mitigate the impact of environmental stress [128]. 

## 4. Perspectives

Novel protein sources are functional and may exert a positive modulatory effect in fish subjected to chronic stress. Fish growth can be promoted by inhibiting cortisol hormone, potentializing antioxidant capacity, and reducing metabolic stress markers with adequate nutrition. More studies are needed to achieve the level of inclusion for each species.

Animal by-product meals are rendered products from farm animals that can be used as valuable feedstuffs for fish feed, displacing traditional fishmeal and promoting a circular economy system by reducing waste and reintroducing them in the economy as high-value products. These ingredients have shown positive effects in fish under stress conditions. Yeast hydrolysate is rich in peptides and is used as an additive because it stimulates the antioxidant system in fish and prepares the organism’s defense to cope with infections. Including yeast hydrolysate in the diet might prevent outbreaks when fish are subject to stress. The fungal meal significantly affects dietary interventions and is a promising feed source that can be added to animal feed to replace familiar protein sources such as fishmeal. Valuable filamentous fungal biomass contains high protein concentrations composed of functional amino acids. Mushrooms and mycelia provide protein, lipids, fatty acids, vitamins, and antioxidants. However, research on using novel ingredients for dietary interventions has been performed in most acute rather than chronic stress situations.

Insects are one of the most promising alternative ingredients to supply a protein source. Their nutritional composition includes long-chain polyunsaturated fatty acids (VLPUFAs), Vitamin C, and Vitamin D, which promote immune stimulation, and fatty acids that allow fish maintenance besides environmental stress. Then, insects can be used because of their high amount of protein and their beneficial effects on fish health. Interestingly, amino acids (e.g., Trp and Phe), vitamins E and C, and some minerals (e.g., Se) are functional nutrients that alleviate abiotic chronic stress. More studies must be performed to evaluate novel meal ingredients to optimize the nutritional composition of fish feeding. On the other hand, investigations must be performed to establish the level of inclusion by species. Additionally, the combination and pretreatment of ingredients, such as hydrolysates, solid fermentations, and metabolites extraction, potentialize the effects of feeding for chronically stressed welfare and, not less importantly, the performance of aquaculture production systems. Therefore, more studies are needed to elucidate this novel generation of diets aiming to accomplish sustainable and resilient aquaculture.

## 5. Conclusions

Imminent intensive production, global warming, and the severity of infections directly affect fish integrity under farming conditions. Abiotic stress takes visibility due to negative impacts on the organism, resulting in diminished production. Novel ingredients for protein sources possess functional nutrients that promote a set of fish responses to cope with adverse environmental conditions. The frontier of science in fish nutrition is focused on developing a novel generation of diets to make aquaculture more resilient from molecular, individual, and shoal levels, with benefits to livestock units.

## Figures and Tables

**Table 1:** Terms used in literature to refer to abiotic stressors.

Stressors	Terminology	Reference
Netting, confinement, and flashlight	Chronic stress protocol	[25]
Temperature, pH, turbidity, toxicants, pathogens, predators, handing	Environmental factors	[14]
Temperature	Water quality variables	[27]
Cold current, high temperature, low precipitation, ammonia, harmful algal blooms, warming	Abiotic factor	[28]
Chemicals (insecticides, pesticides), dissolved oxygen, pH, temperature	Abiotic factor	[11]
Temperature, chemical contamination, photoperiod, salinity	Abiotic factor	[29]

## Data Availability

Not applicable.
